# Harpalycin 2 inhibits the enzymatic and platelet aggregation activities of PrTX-III, a D49 phospholipase A_2_ from *Bothrops pirajai* venom

**DOI:** 10.1186/1472-6882-12-139

**Published:** 2012-08-27

**Authors:** Rafael M Ximenes, Renata S Alves, Ticiana P Pereira, Renata M Araújo, Edilberto R Silveira, Marcelo M Rabello, Marcelo Z Hernandes, Veronica C G Soares, Daniel Bristot, Camila L Pires, Daniela O Toyama, Henrique H Gaeta, Helena S A Monteiro, Marcos H Toyama

**Affiliations:** 1 Department of Physiology and Pharmacology, Federal University of Ceará, UFC, Rua Coronel Nunes de Melo 1127, Fortaleza, CE, 60430-270, Brazil; 2 Department of Clinical and Toxicological Analysis, Federal University of Ceará, UFC, Rua Capitão Francisco Pedro 1210, Fortaleza, CE, 60430-370, Brazil; 3 Department of Chemistry, Federal University of Rio Grande do Norte, UFRN, Av. Senador Salgado Filho 3000, Natal, RN, 50078-970, Brazil; 4 Department of Organic and Inorganic Chemistry, Federal University of Ceará, UFC, Campus do Pici, Bloco 940, PO Box 12200, Fortaleza, CE, 60455-760, Brazil; 5 Department of Pharmaceutical Sciences, Federal University of Pernambuco, UFPE, Av. Arthur de Sá, s/n, Recife, PE, 50740-520, Brazil; 6 Department of Biochemistry, Institute of Biology, State University of Campinas, UNICAMP, Rua Monteiro Lobato 255, Campinas, SP, 13082-862, Brazil; 7 São Vicente Unit, State University of São Paulo Júlio Mesquita Filho, UNESP, Praça Infante Dom Henrique, s/n, São Vicente, SP, 11330-900, Brazil; 8 Center of Biological and Health Sciences, Mackenzie Presbyterian University, Rua da Consolação 896, São Paulo, SP, 01302-970, Brazil

**Keywords:** PrTX-III, Phospholipase A_2_, *Bothrops pirajai*, *Harpalyce brasiliana*, Isoflavone

## Abstract

**Background:**

Harpalycin 2 (HP-2) is an isoflavone isolated from the leaves of *Harpalyce brasiliana* Benth., a snakeroot found in northeast region of Brazil and used in folk medicine to treat snakebite. Its leaves are said to be anti-inflammatory. Secretory phospholipases A_2_ are important toxins found in snake venom and are structurally related to those found in inflammatory conditions in mammals, as in arthritis and atherosclerosis, and for this reason can be valuable tools for searching new anti-phospholipase A_2_ drugs.

**Methods:**

HP-2 and piratoxin-III (PrTX-III) were purified through chromatographic techniques. The effect of HP-2 in the enzymatic activity of PrTX-III was carried out using 4-nitro-3-octanoyloxy-benzoic acid as the substrate. PrTX-III induced platelet aggregation was inhibited by HP-2 when compared to aristolochic acid and p-bromophenacyl bromide (p-BPB). In an attempt to elucidate how HP-2 interacts with PrTX-III, mass spectrometry, circular dichroism and intrinsic fluorescence analysis were performed. Docking scores of the ligands (HP-2, aristolochic acid and p-BPB) using PrTX-III as target were also calculated.

**Results:**

HP-2 inhibited the enzymatic activity of PrTX-III (IC_50_ 11.34 ± 0.28 μg/mL) although it did not form a stable chemical complex in the active site, since mass spectrometry measurements showed no difference between native (13,837.34 Da) and HP-2 treated PrTX-III (13,856.12 Da). A structural analysis of PrTX-III after treatment with HP-2 showed a decrease in dimerization and a slight protein unfolding. In the platelet aggregation assay, HP-2 previously incubated with PrTX-III inhibited the aggregation when compared with untreated protein. PrTX-III chemical treated with aristolochic acid and p-BPB, two standard PLA_2_ inhibitors, showed low inhibitory effects when compared with the HP-2 treatment. Docking scores corroborated these results, showing higher affinity of HP-2 for the PrTX-III target (PDB code: 1GMZ) than aristolochic acid and p-BPB. HP-2 previous incubated with the platelets inhibits the aggregation induced by untreated PrTX-III as well as arachidonic acid.

**Conclusion:**

HP-2 changes the structure of PrTX-III, inhibiting the enzymatic activity of this enzyme. In addition, PrTX-III platelet aggregant activity was inhibited by treatment with HP-2, p-BPB and aristolochic acid, and these results were corroborated by docking scores.

## Background

Medicinal plants have been used in traditional medicine as an alternative and supplementary therapy to treat snakebite poisoning, and many other health problems, such as inflammatory disorders [1]. In 1982, Nakagawa et al. [2] tested the anti-ophidian activity of “Específico Pessoa”, a Brazilian phytotherapic tincture used in folk medicine to treat snakebite, mainly in the Amazon region. Two prenylated pterocarpans, the cabenegrins A-I and A-II, had been pointed out as responsible for this activity. Several years later, the same compounds were found in *Harpalyce brasiliana* Benth (Papilionoideae), popularly known in the Northeast of Brazil as “raiz-de-cobra” (Port. Lit.: snakeroot). Its roots have been used to treat snakebite [3], while its leaves are claimed to be anti-inflammatory [Personal ethnopharmacological survey].

Secretory phospholipases A_2_ (sPLA_2_) are present in most snake venoms and show important neurotoxic and myotoxic activities, and most of them are not fully neutralized by commercial antivenom sera [4]. Flavonoids exhibit different inhibitory levels in group I sPLA_2_s from porcine pancreas and *Naja naja* venom, and in group II sPLA_2_s from *Vipera russelii* and *Crotalus atrox* venoms. The most important regions involved in the inhibition of sPLA_2_ have been reported to be the hydroxyl groups at 30- and 40-positions [5,6]. Iglesias et al. [7] showed that flavonoids such as morin can modify the secondary structure of the snake venom sPLA_2_. Toyama et al. [8] showed that 7-hydroxycoumarin interacts with sPLA_2_ and causes some structural modifications, indicating its potential use to suppress inflammation induced by sPLA_2_.

Group II sPLA_2_ enzymes have been found in inflammatory sites in animal models, as well as in synovial fluids from patients with rheumatoid arthritis and a number of inflammatory diseases, in which, a correlation between serum sPLA_2_ levels and disease activity has been observed [9,10]. Exogenous administration of sPLA_2_, such as snake venom sPLA_2_, induces and/or exacerbates inflammatory response in animals [11,12]. Structural analyses revealed that snake venom sPLA_2_s have a similar molecular profile to those of human secretory PLA_2_s as well as a conserved catalytic site [13], thus making them useful tools for the search of new anti-phospholipase A_2_ drugs.

Pterocarpans have been pointed out as possible compounds involved in snakebite protection of “Específico Pessoa”. These molecules are characterized as a group of isoflavonoids formed from isoflavones. Here, the anti-phospholipasic activity of harpalycin 2 (HP-2), an isoflavone isolated from the leaves of *Harpalyce brasiliana* Benth., against PrTX-III was investigated. Aristolochic acid and p-bromophenacyl bromide were used as gold standards sPLA_2_ inhibitors. PrTX-III is a catalytically active, hemolytic and platelet aggregant D49 sPLA_2_, isolated from the *Bothrops pirajai* venom [14].

## Methods

### Venom

*Bothrops pirajai* venom was purchased from Bio-Agents Serpentarium in the city of Batatais (São Paulo, Brazil).

### Plant material

Leaves of *Harpalyce brasiliana* Benth. were collected at the Chapada do Araripe, Barbalha (Ceará, Brazil) by Prof. Edilberto Rocha Silveira. Botanical authentication was made by Prof. Edson P. Nunes of the Department of Biology, Federal University of Ceará. Voucher specimen (number: 32 525) has been deposited at the Prisco Bezerra Herbarium (EAC), Department de Biology, Federal University of Ceará, Fortaleza (Ceará, Brazil).

### General procedures

The mass spectra were obtained on a Hewlett-Packard 5971 mass spectrometer by electron impact ionization (70 eV). ^1^ H and ^13^C NMR spectra were recorded on a Bruker Avance DRX-500 (500 MHz for 1 H and 125 MHz for 13C); chemical shifts were expressedin scale and were referenced to residual DMSO (2.5 and 39.5 ppm). Silica Gel 60 (Merck, 70–230 mesh) was used for analytical TLC. Column chromatographies were performed over silica gel (Merck, 60 F254 230–400 mesh).

### Extraction and isolation of harpalycin 2

Leaves of *Harpalyce brasiliana* were pulverized and extracted with EtOH at room temperature. The solvent was removed under reduced pressure which produced a dark viscous extract (HBFE). Liquid-liquid partition of a water suspension of HBFE (110 g) using petrol ether, CHCl_3_, EtOAc and n-BuOH yielded five fractions after solvent evaporation or lyophilization: HBFEEp (24.5 g), HBFEC (22.4 g), HBFEA (6.8 g), HBFEB (30.4 g) and HBFEAq (21.2 g).

Flash chromatography of HBFEC (12.0 g) using n-hexane and EtOAc as binary mixtures of increasing polarity afforded 30 fractions, which were pooled in 9 fractions after thin layer chromatography (TLC) analysis. HBFEC (10–12) presented a yellow precipitate, yielding 120.0 mg of a white amorphous solid (m.p. 206.9-208.9°C). Spectrometric analysis showed the structure of the isoflavone harpalycin 2. The fractions HBFEC (8–9) and HBFEC (13–17) were purified, using the same method, yielding more 200.0 mg of harpalycin 2 (HP-2).

### Purification of PrTX-III

*Bothrops pirajai* venom was first fractioned in two consecutive chromatographic steps as described by Toyama et al. [15]. Approximately 20 mg of the lyophilized venom was dissolved in 250 μL of 0.05 M ammonium bicarbonate, pH 7.8 (Buffer A). After homogenization, venom solution was clarified by centrifugation at 10,000 rpm for 3 min. The supernatant was inserted into a Protein Pack SP 5PW column (0.78 x 7.0 cm) and the elution performed using a linear gradient of concentration between 0.05 and 1.0 M ammonium bicarbonate with 750 μL of 0.1% (v/v) trifluoroacetic acid (Buffer B). The fractions were collected, lyophilized and clarified by centrifugation and the supernatant inserted into a μ-Bondapack C18 column (0.78 cm x 30 cm) (Waters 991-PDA system). Elution of peaks proceeded with a linear gradient between 0% and 66.5% (v/v) acetonitrile (solvent B) in 0.1% (v/v) trifluoroacetic acid, at a flow rate of 2.0 mL/min. Absorbances were monitored at 280 nm. Fractions were collected, lyophilized and stored at −20°C. The PrTX-III fraction was identified by the retention time and the measurement of the catalytic activity (since PrTX-III is the activity isoform of *B. pirajai* venom). The purity degree of PrTX-III was evaluated by Tricine SDS-PAGE and by mass spectrometry on a MALDI-TOF mass spectrometer, as previously described [12,16].

### Measurement of sPLA_2_ activity

sPLA2 activity was measured following the protocols described by Lee et al. [13] and modified by Toyama et al. [12] for 96-well plate, using 4-nitro-3-octanoyloxy-benzoic acid (4N3OBA, manufactured by BIOMOL, USA) as the substrate. Enzyme activity was calculated based on the increase in absorbance after 20 min. All assays were performed using n = 12 and absorbances at 425 nm were measured using a SpectraMax 340 multiwell plate reader (Molecular Devices, Sunnyvale, CA). After the addition of sPLA_2_ (20 μg), the reaction mixture was incubated for 40 min at 37°C, and the absorbance read at 10 min intervals. For the estimation of the IC_50_ of harpalycin 2 for PrTX-III, different concentrations of HP-2 (5, 10, 20, 40 and 80 μg) were added to each well. The remaining enzymatic assay was conducted as described above. Harpalycin 2 was previously dissolved in DMSO 1%.

### Incubation of sPLA_2_ with harpalycin 2 and purification of HP-2 treated sPLA_2_ (PrTX-III: HP-2) and amino acid analysis

The incubation of sPLA_2_ with HP-2 (w: w; 4:1), followed the procedures described by Iglesias et al. [7]. Briefly, HP-2 was dissolved in DMSO 1%. 250 μL of HP-2 solution was added to 1,000 μL of homogenized solution of PrTX-III. The mixed solution was incubated for 60 min in a water bath at 37°C. Samples of 200 μL of this mixture were loaded into a preparative reverse phase HPLC column to separate the treated enzyme (PrTX-III: HP-2) from HP-2. After column equilibration with buffer A (aqueous solution of 0.1% TFA), samples were eluted using a discontinuous gradient of buffer B (66.6% of acetonitrile in 0.1% TFA) at a constant flow rate of 2.0 mL/min. The chromatographic run was monitored at 214 nm for detection of PrTX-III, PrTX-III: HP-2 and HP-2. 1 nmol of purified protein (PrTX-III or PrTX-III: HP-2) was hydrolyzed with 6 N HCl (200 μL) in the presence of 10μL of phenol solution for prevention of unspecific amino acid oxidation. Amino acid hydrolysis was performed at 106°C for 24 h. After this time, the excess HCl was removed and the hydrolyzed amino acids were rehydrated with a solution of ethanol: water: triethylamine (v: v; 2:2:1). Post-column derivatization was performed with an aqueous solution of phenylisothiocyanate (ethanol: water: triethylamine: phenylisothiocyanate; v: v; 7:1:1:1). Samples and amino acid standards were derivatized using a PICO-TAG amino acid analyzer system (Waters, USA).

### Molecular exclusion chromatography

A molecular exclusion chromatography was initially performed using an AP-1 column (Waters, 1x60 cm) previous packed with Superdex 75 (GE Healthcare Pharmacia) as previously described by Oliveira et al. [17]. For molecular exclusion chromatography of the PrTX-III and PrTX-III: HP-2 as well as the protein marker, 1 mg of the protein sample was dissolved in the same buffer used for equilibration of the chromatographic column and sample elution (Potassium phosphate buffer 0.05 M, pH 7.5). Samples were dissolved in 250 μL of this buffer and then centrifuged at 4,500 *g* for 5 minutes. 200 μL of supernatant was recovered and taken at 37°C for 60 minutes before injection into the column. Injections of 25 μL of each sample were carried out through the column; and elution of fractions was performed under isocratic condition with constant flow rate of 0.2 mL/min and monitored at 280 nm.

### Mass spectrometry

The molecular mass of PrTX-III and PrTX-III: HP-2 were determined by matrix-assisted laser desorption ionization-time-of-flight (MALDI-TOF) mass spectrometry using a Voyager-DE PRO MALDI-TOF mass spectrometer (Applied Biosystems®, Life Technologies^TM^, USA). One microliter of samples (PrTX-III and PrTX-III: HP-2) in 0.1% TFA was mixed with 2 μL of the matrix a-cyano-4-hydroxycinnamic acid, 50% acetonitrile, and 0.1% TFA (v/v). The matrix was prepared with 30% acetonitrile and 0.1% TFA (v/v). The equipment conditions were as follows: accelerating voltage of 25 kV, laser fixed at 2,890 μJ/com2, delay of 300 ns and linear analysis mode.

### Circular dichroism spectroscopy

Purified enzymes - native and HP-2 treated PrTX-III – were dissolved in a 10 mM sodium phosphate buffer (pH 7.4) and the final protein concentrations were adjusted to 8.7 mM. After centrifugation at 4,000 *g* for 5 min, samples were transferred to a 1 mm pathlength quartz cuvette. Circular dichroism spectra in the wavelength range of 185–300 nm were acquired in-house with a J720 spectropolarimeter (Jasco^©^, Japan) using a bandwidth of 1 nm and a response time of 1 s. Data collection was performed at room temperature with a scanning speed of 100 nm/min. Nine scans were taken for each sample and all spectra were corrected by subtraction of buffer blanks.

### Intrinsic fluorescence

The relative intrinsic fluorescence intensity of native PrTX-III or HP-2 treated PrTX-III (PrTX-III: HP-2) was monitored with a spectrofluorimeter (Shimadzu^©^, Japan). 2.0 mL of the reaction mixtures, consisting of 100 mM Tris–HCl buffer (pH 7.4), sPLA_2_ (200 μg/mL) and 5 mM CaCl_2_, were put into a 10 mm pathlength quartz cuvette. Fluorescence was measured at between 300 and 450 nm after excitation at 280 nm.

### Platelet aggregation studies

The platelet aggregation activities were conducted as described by Oliveira et al. [18] and dos Santos et al. [19]. Venous blood was collected with informed consent from healthy volunteers who formally denied taking any medication in the previous 14 days. All experiments using human material were carried out according to Helsinki Declaration and were approved by the Ethical Committee for Human Research of State University of Campinas under the no. 0323.0.1.146.000-09. Blood was collected by a two-syringe technique using polypropylene syringes and 19-gauge needles, and immediately transferred into polypropylene tubes containing 1/10 of final volume of 3.8% trisodium citrate. After removing the platelet-rich plasma (PRP), the remaining blood was prepared by centrifugation at 200 *g* for 10 min and the washed platelet solution (WP) was obtained from the residue by centrifugation of citrated blood at 1,500 *g* for 20 min. The platelets were left for 1 hour at room temperature to recover their sensitivity to aggregating agents. Platelet counts were performed on a Coulter S Plus (Coulter Electronics, Hialeah, FL) or by phase-contrast microscopy. Platelet aggregation was carried out using 400 μL of the washed platelets solution in a cuvette and kept at 37°C with constant stirring. The desired concentration of protein was added and 3 minutes after to the addition, the aggregation was recorded for 5–10 min by using an aggregometer (Payton Scientific Inc., USA). Aggregation experiments were performed with 10 μg of PrTX-III and PrTX-III: HP-2., PrTX-III: aristolochic acid or PrTX-III: p-bromophenacyl bromide. In order to elucidate HP-2 mode of action, 10 μL of HP-2 (5 mg/mL), AACOCF3 (1 mM) and INDO (1 mM) were added 5 minutes before the addition of native PrTX-III (10 μg) or arachidonic acid (AA, 50 mM).

### Docking studies

The structural optimizations of the harpalycin 2 (HP-2), aristolochic acid (Aris Ac) and p-bromophenacyl bromide (p-BPB) ligands were initially achieved using the AM1 method [20] implemented in the BioMedCache program [21] with default values for the convergence criteria. Docking calculations were performed with the GOLD 4.0 program [22] to obtain the *in silico* affinity of the ligands with respect to the PrTX-III target. The tridimensional coordinates of the target were taken from the RCSB Protein Data Bank (PDB), under the PDB code 1GMZ, as a dimeric quaternary structure. The “A” chain was chosen for all the calculations. The docking calculations were performed to consider the flexibility of all the ligands and the flexibility of the target, in order to represent the induced fit generated by the presence of non-native ligands, using the following approach: the residues Phe5, Ile9, Phe18, Tyr21, Val22, Tyr27, His47, Asp48, Lys60 and Phe96 were configured in such a way that their side-chain torsions were considered active during the calculations. The active site was defined as all the atoms within a radius of 8.0 Å from the co-crystallized isopropyl alcohol (IPA).

### Statistical analysis

Results were expressed as mean ± S.E.M. and analyzed by ANOVA followed by Dunnett’s test using GraphPad Prism® 5.0 with significance set at p < 0.05*.

## Results

**Figure 1:**
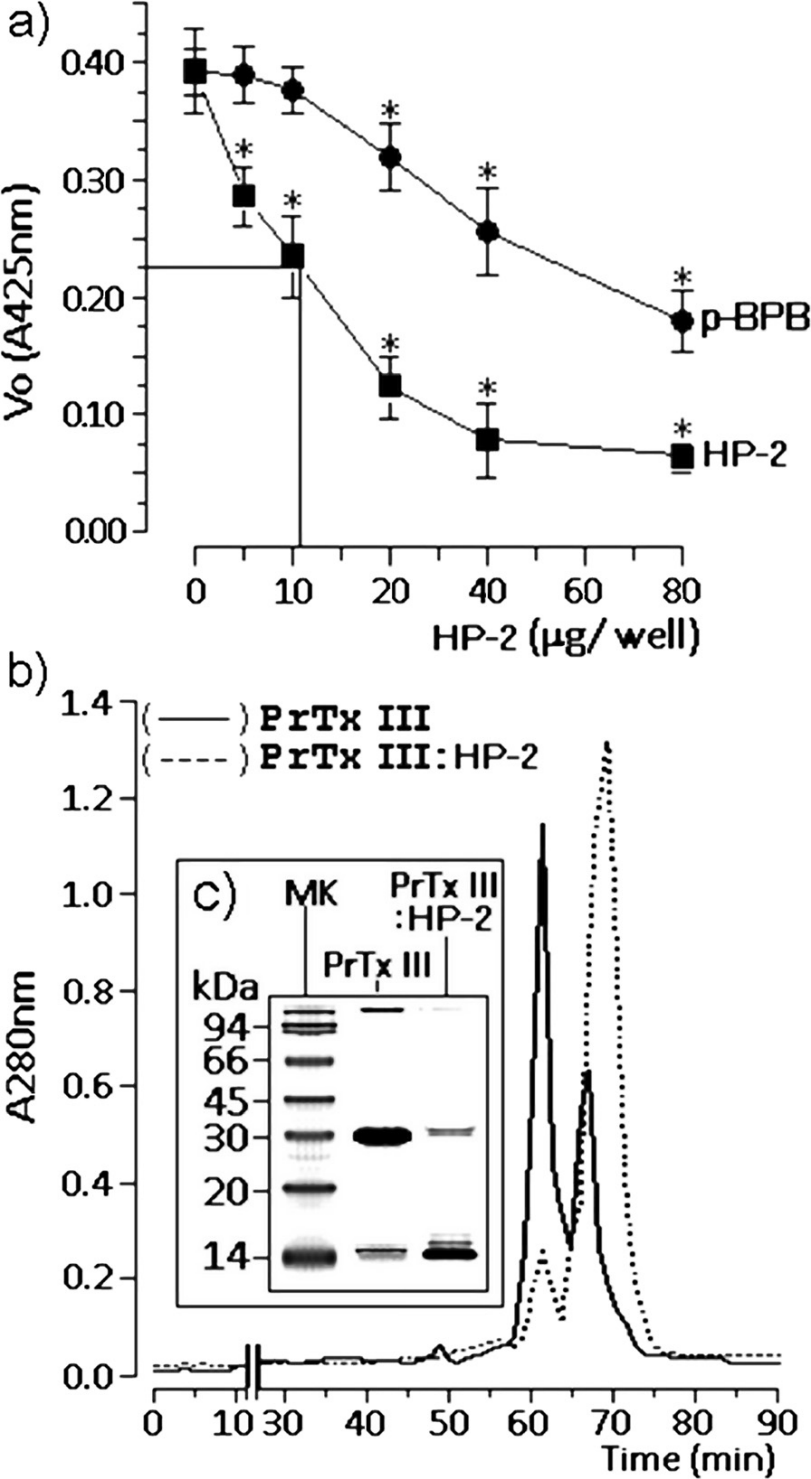
**a. Demonstrates the inhibitory effect induced by p-BPB and HP-2 on the enzymatic activity of PrTX-III in presence of 4-nitro-3-octanoyloxy-benzoic acid as substrate of the enzymatic reaction.** Phospholipasic A_2_ activity was expressed as Vo at 425 nm. Each point represents the mean ± SEM (n = 12) and *p < 0.05. **b.** Shows the molecular exclusion chromatography of the PrTX-III and PrTX-III previously treated with HP-2 (PrTX-III: HP-2). Both samples were subjected to the same chromatographic run conditions. **c.** Displays the Tricine SDS-PAGE profile where the MK was the low molecular weight marker.

Harpalycin 2 (HP-2) was isolated as a white amorphous solid with m.p. 232.6-234.4°C. Its molecular formula of C_21_H_18_O_7_ was established by the molecular ion at *m/z* 382 Daltons in the MS spectrum. Structure elucidation was performed by spectroscopic means, including 1D and 2D NMR, and comparison with the data from literature [3]. Harpalycin 2 (HP-2) showed a potent inhibitory capacity when compared to the classical sPLA_2_ inhibitor p-bromophenacyl bromide (p-BPB), with an IC_50_ calculated at 11.34 ± 0.28 μg per well, whereas p-BPB showed only a marginal inhibition of PrTX-III catalytic active (Figure 1a). The isolation of PrTX-III yielded 15% (w: w). Native PrTX-III subjected to molecular mass exclusion chromatography showed the presence of a main fraction with a molecular mass that was estimated at 25,000 Da and a minor fraction with molecular mass estimated at 14,000 Da. The chromatographic profile of PrTX-III: HP-2 revealed the presence of a main fraction, which had its molecular mass estimated at 14,000 Da, and a minor fraction with molecular mass of 20,000 Da. According to our data, the main fraction eluted from the sample of PrTX-III: HP-2 represented approximately 90% of the total. These results showed that HP-2 treatment of PrTX-III induced a reduction of the dimerization of PrTX-III, while the native PrTX-III showed a typical transition from the monomer to dimmer (Figure 1b). The chromatographic results were also corroborated using a Tricine SDS-PAGE analysis (Figure 1c).

**Figure 2:**
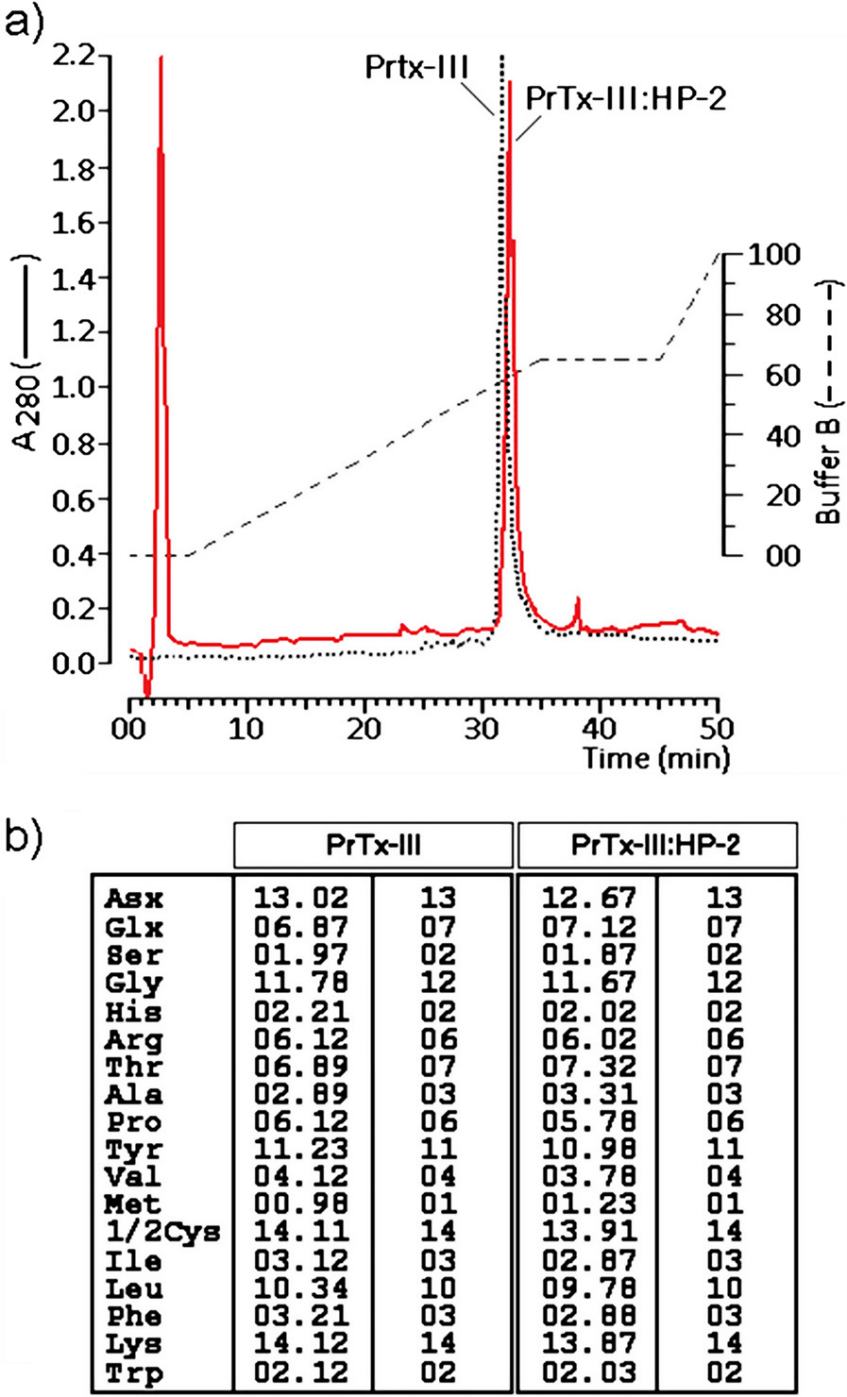
**a. Shows the reverse phase HPLC profile of the PrTX-III and the PrTX-III from the incubation with HP-2.** Both samples were eluted at same chromatographic conditions using a linear discontinuous increasing of buffer **b.** The amino acid analysis of PrTX-III and PrTX-III: HP-2. The amino acid counting was expresses as number of each amino acid residues found by each mol of protein.

The reverse phase HPLC profile of native PrTX-III indicated that sPLA_2_ fraction was eluted at 31.7 minutes whereas PrTX-III: HP-2 was eluted at 32.3 minutes, showing a discreet shift in the retention time of this protein (Figure 2a). The amino acid analysis of native and PrTX-III previously treated with HP-2 did not revealed changes in the amino acid amount in both samples (Figure 2b). Mass spectrometry data from the native PrTX-III and PrTX-III: HP-2 showed masses of 13,750.3 Da and 13,736.2 Da, respectively. These results suggest that binding of the HP-2 with PrTX-III did not involve the formation of stable complexes. The discrepancy between results from the amino acid analysis and spectrometric studies is probably due to the presence of HCl 6 N used for rupturing the amino acid bond that could revert the acid condition of some amino acids or restore the H^+^ lost in the reaction with HP-2 with PrTX-III. This is true for the PICO-TAG amino acid analysis based in the post column derivatization and the final results also did not differentiate Asp from Asn or Glu from Gln.

**Figure 3:**
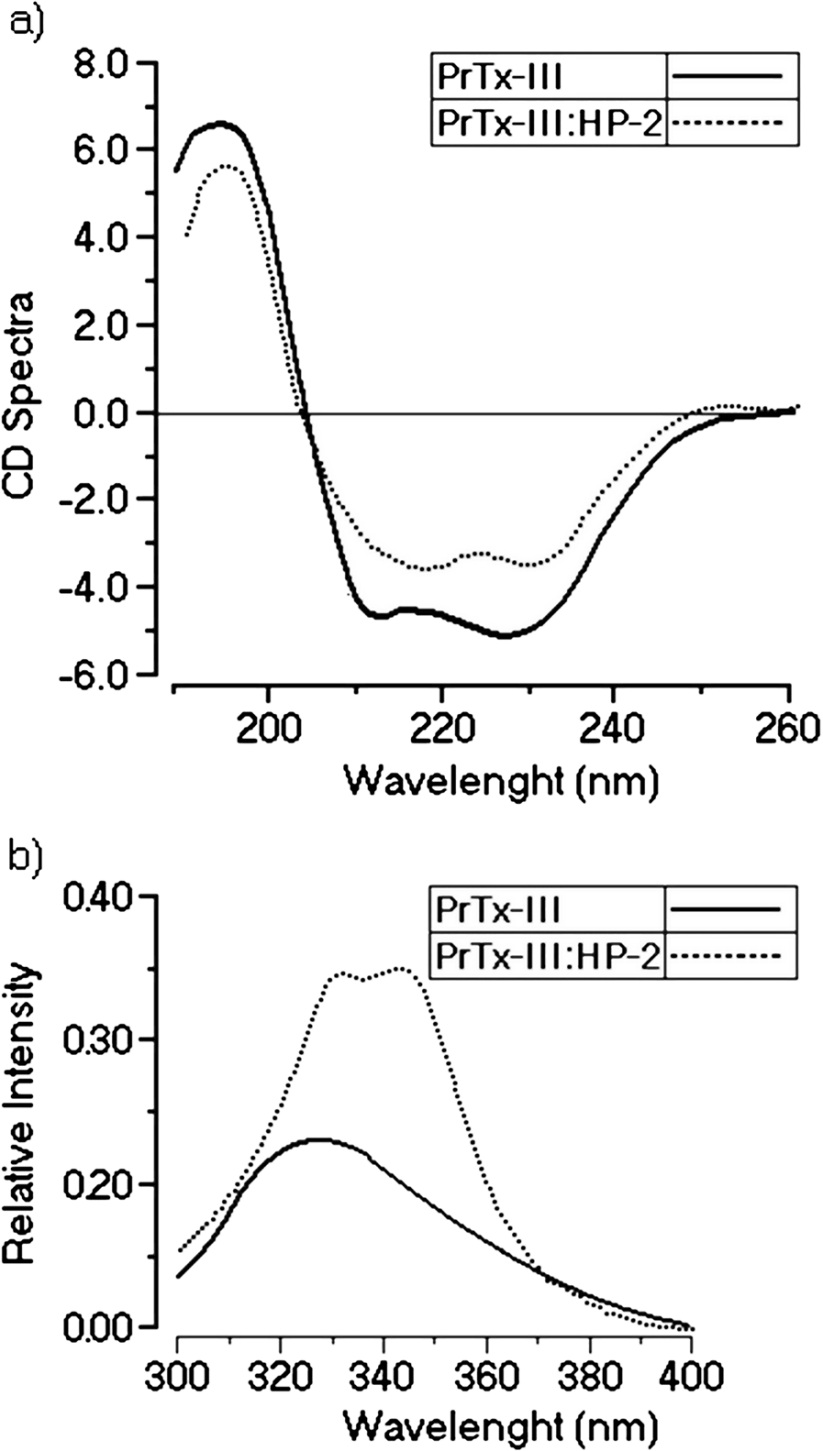
**a. Displays the CD spectra of native PrTX-III and PrTX-III treated with HP-2. Data over the range 185–260 nm is shown and CD spectra are expressed in theta machine units in millidegrees.b.** Shows the fluorescence curves of untreated sPLA_2_ (PrTX-III) and HP-2 treated sPLA_2_ (PrTX-III: HP-2).

The CD spectra of the PrTX-III: HP-2 showed an evident decrease of total alpha-helix as well as an increase of beta-sheets when compared with the spectra of native PrTX-III, suggesting a partial unfolding of the protein induced by HP-2 treatment (Figure 3a). This data was reinforced by the increase in the tryptophan fluorescence of the treated protein as seen in Figure 3b.

**Figure 4:**
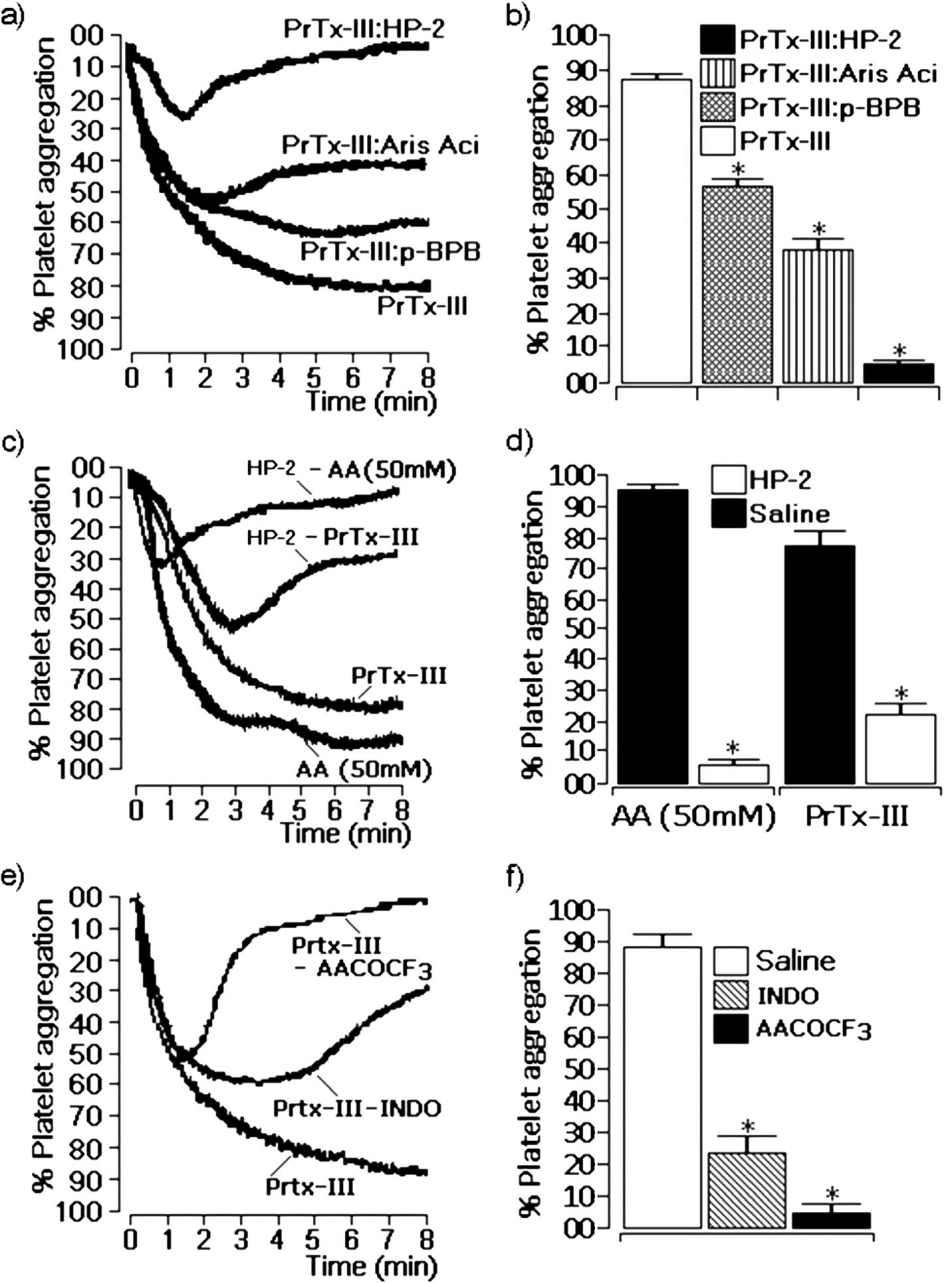
**Aggregation assays were performed using a protein concentration of 10 μg of native PrTX-III or PrTX-III previously treated with harpalycin 2 (HP-2), aristolochic acid (Aris Ac) or p-bromophenacyl bromide (p-BPB)** (**a**). The effect of previous incubation of platelets with HP-2 before the administration of PrTX-III or arachidonic acid is showed in (**c**). Previous incubation with AACOCF3 or INDO before the administration of PrTX-III is showed in (**e**). (**b**), (**d**) and (**f**) demonstrate the platelet aggregation results after 8 minutes of experimental condition and each point in the respective table represent the mean ± SEM (n = 4) and *p < 0.05.

The platelet aggregation observed for native PrTX-III was blocked by treatment of PrTX-III with HP-2, and partially inhibited by the treatment with aristolochic acid (Aris Ac) p-BPB (Figure 4a). Figure 4a also shows that the PrTX-III treated with HP-2 was able to trigger an initial platelet aggregation of approximately 20%, which was rapidly reverted. This effect was not very evident for the treatment of PrTX-III with aristolochic acid. HP-2 previously added to the platelets inhibited the aggregating effect induced by arachidonic acid as well as the effect induced by PrTX-III (Figure 4b). The effect of HP-2 was similar that of AAOCF3 (arachidonyl trifluoromethyl ketone), a specific PLA_2_ inhibitor. Previous treatment with indomethacin only partially inhibited the aggregation induced by PrTX-III (Figure 4c), showing that the phospholipase A_2_ activity is crucial to full platelet aggregation but that the PLA_2_ downstream cascade is also involved, which was corroborated by the fact that HP-2 also inhibited arachidonic acid induced platelet aggregation.

**Figure 5:**
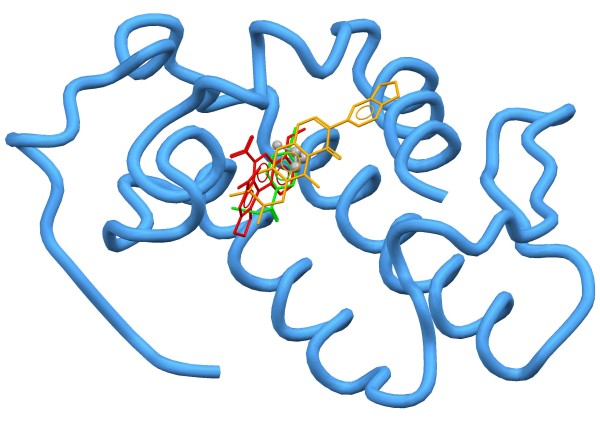
**Superposition of the best docking solution for the ligands harpalycin 2, aristolochic acid and p-bromophenacyl bromide (stick models in orange, red and green colors, respectively).** The co-crystallized isopropyl alcohol (IPA) is showed as a gray ball model. The hydrogen atoms were omitted for clarity reasons.

**Figure 6:**
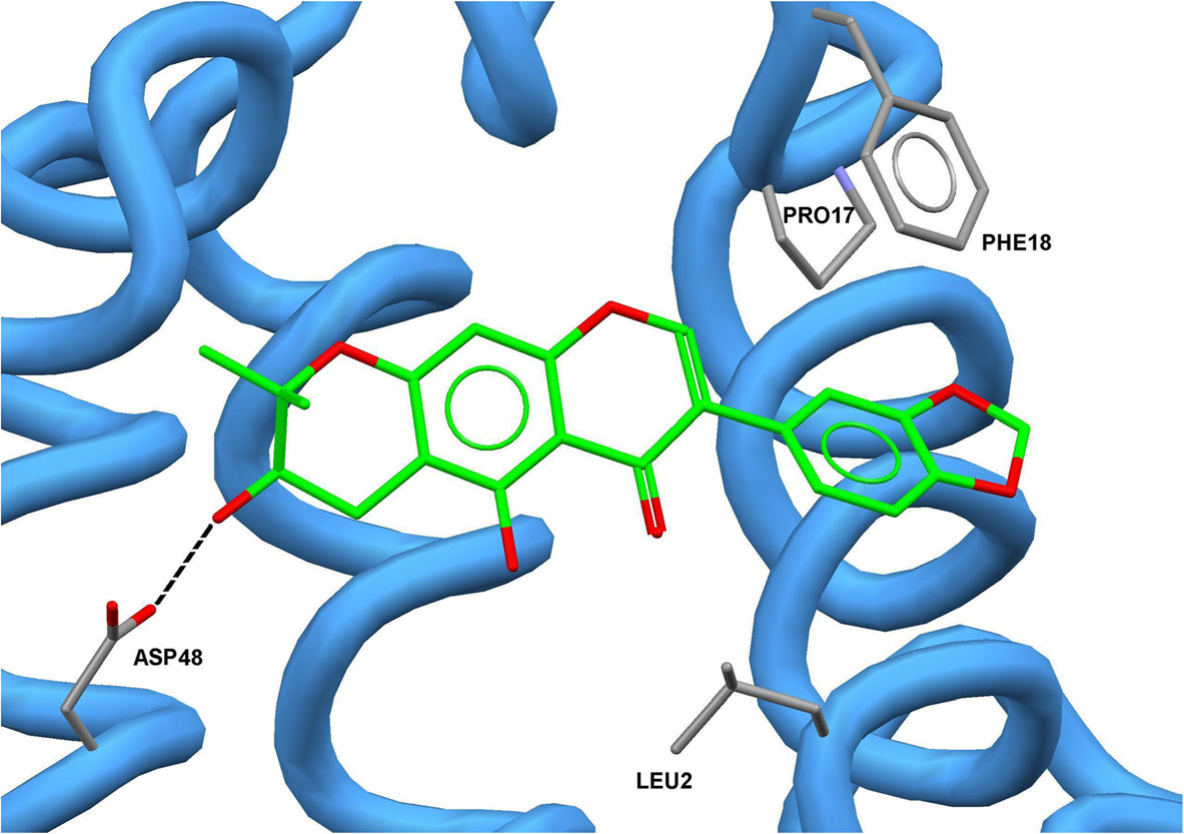
**Detailed view of the best docking solution for the harpalycin 2 ligand.** The hydrogen bonds are showed in black lines. The hydrogen atoms were omitted for clarity reasons.

The superposition of the best docking solutions for the harpalycin 2 (HP-2), aristolochic acid and p-bromophenacyl bromide ligands can be observed in Figure 5. The docking scores for these calculations were 36.78, 35.29 and 26.63, respectively, showing a better affinity between the PrTX-III target and harpalycin 2, in comparison with aristolochic acid and p-bromophenacyl bromide. A detailed inspection of the binding mode of HP-2 in PrtX-III can be found in Figure 6. The molecular reasons for the greater stability (greater docking score) of the harpalycin 2 ligand in the active site of the PrTX-III can be explained mainly by the presence of some important intermolecular interactions, in particular, a hydrogen bond that harpalycin 2 establishes with the residue Asp48, with 2.88 Å (see Figure 6). The detailed comparison between the docking results obtained for these three ligands can be found in Table 1.

**Table 1:** Details of the docking results and the intermolecular interactions identified for the harpalycin 2 (HP-2), aristolochic acid (Aris Ac) and p-bromophenacyl bromide (p-BPB) ligands with the PrTX-III target

	**HP-2**		**Aris Ac**		**p-BPB**	
inhibition % of platelet aggregation	**95**		**55**		**35**	
GOLD score	**36.78**		**35.29**		**26.63**	
Residues	**HB**	**HP**	**HB**	**HP**	**HB**	**HP**
LEU2	−	yes	−	−	−	−
PHE5	−	−	−	yes	−	yes
ILE9	−	−	−	yes	−	yes
PRO17	−	yes	−	yes	−	−
PHE18	−	yes	−	−	−	−
TYR21	−	−	−	yes	−	yes
ASP48	2.88	−	−	−	−	−
PHE96	−	−	−	yes	−	yes

The inhibition % of platelet aggregation data was included for comparison reasons.

* HB stands for Hydrogen Bonds and HP for Hydrophobic Interactions.

** All HB distance values are given in Å.

## Discussion

Snake venoms are rich sources of phospholipase A_2_ and PLA_2_-homologues, active calcium-binding Asp49 enzymes and essentially inactive Lys49 proteins, respectively. They are responsible for multiple pharmacological effects, some of which are dependent on catalytic activity and others of which are not. The pharmacological or biological effects that do not depend on enzymatic activity are driven by other pharmacological regions or sites that include calcium-binding loop, beta-wing and C-terminal region [16,19,23,24]. The precise location or mapping of these pharmacological sites is not easy to find due to the fact that the residues involved in myotoxicity and neurotoxicity significantly overlap, suggesting that multiple biological effects observed in many snake venom PLA_2_s are a consequence of superposed structural determinants on the protein surface [24]. However, the consensual idea is that enzymatic activity of PLA_2_ is the major factor responsible for the majority of the pharmacological activity induced by the PLA_2_ in snake venom, such as inflammatory activity, especially due to the arachidonic acid cleavage [25]. PrTX-III showed a sigmoidal behavior on the substrate concentration, which implies cooperation of substrate binding. This effect has been described for other sPLA_2_ such as those isolated from *Bothrops jararacussu*[26] and *Crotalus durissus terrificus*[17] venoms. The transition between the monomers to dimmers increases the phospholipasic A_2_ activity 100 times, which could explain in part the inhibition of PrTX-III by HP-2, since the treatment with HP-2 showed an increase of the monomer form over the dimmeric form.

Crystallographic studies showed that PrTX-III has putative dimmer interface identified in the crystal lattice which brings together the calcium-binding loops of neighboring molecules, along with the C-terminal regions which are disulfide bonded to those loops, thereby offering a possible route of communication between active sites. The interaction of HP-2 with PrTX-III seems to induce a chemical modification of crucial amino acid residues involved in the sPLA_2_ catalysis. It could also modify some important residues related to the dimmer formation, agreeing in CD spectra and fluorescence measurement which suggests that HP-2 induced a partial unfolding of PrTX-III. The N-terminal region as well as the calcium-binding loop and the alpha-helix of sPLA_2_ play an important role for the sPLA_2_ catalysis. The modification in these region leads to an irreversible lost of enzymatic activity of sPLA_2_ and/or its ability for binding to cell membrane [27].

PrTX-III induced a platelet aggregation in a dose dependent manner and this effect was abolished by previous treatment with HP-2. At the beginning of the experiment, however, we observed a slight aggregation that was quickly reverted. Previous report by Polgár et al. [28] suggests that a glycophosphatidylinositol-anchored platelet-membrane heparan sulphate proteoglycan is the binding site for sPLA_2_ on platelets. Some specific mutation studies carried out with PLA_2_ have shown that the binding of secretory PLA_2_ to this receptor involves a specific recognition of the CRD region of this receptor, which is located near the catalytic site of PLA_2_ and calcium-binding loop [18]. The binding of HP-2 to the active site of PrTX-III could interfere with this ability. The HP-2 effect on the platelet resembles the effect induced by AACOCF3, which is a selective PLA_2_ inhibitor, showing a potential anti-inflammatory effect of harpalycin 2 that could be derived from PLA_2_ inhibition. Indomethacin partially inhibited the aggregating effect of PrTX-III pointing to the role of cyclooxygenases in this pharmacological action. HP-2 could also being acting in this pathway, since it can block the platelet aggregating effect induced by arachidonic acid, which is a step forward in the downstream cascade of PLA_2_/COX-1/2/eicosanoids.

These experimental results corroborate with the *in silico* observations, where harpalycin 2 ligand presented a greater affinity for the PrTX-III active site, in comparison with the two other ligands (aristolochic acid and p-BPB). This trend implies that the ligands with the best activities have greater stabilities (high docking scores) within the PrTX-III target according to the docking results, and the ligands with the worst activities have weak interactions (low scores) with the same target. These results suggest that the inhibition of platelet aggregation is due to the inhibition of the interaction between platelet substrate and the sPLA_2_.

## Conclusions

Our results of the harpalycin 2 effect on PrTX-III are important as they indicate a possible anti-ophidian and anti-inflammatory activity of this compound and, as well as in part, corroborate the ethnobotanical use that is described for *H. brasiliana* in the Northeast of Brazil. The in silico results also gave us clues about how these molecules interact in the active site of phospholipases A_2_ inhibiting their enzymatic activity.

## Abbreviations

AA: Arachidonic Acid; AACOCF3: Arachidonyl Trifluoromethyl Ketone; Aris Ac: Aristolochic Acid; COX-1/2: Cyclooxygenases-1/2; HP-2: Harpalycin 2; INDO: Indomethacin; p-BPB: p-Bromophenacyl Bromide; PLA_2_: Phospholipase A_2_; PrTX-III: Piratoxin-III; sPLA_2_: Secretory Phospholipase A_2_.

## Competing interests

The authors declare that they have no competing interests to disclose.

## Authors’ contributions

RMX, RSA, TPP, VCGS, DB, CLP and HHG were students who were responsible for the biochemical and pharmacological experiments. MMR and MZH were responsible for the docking studies. RMA and ERS collected the botanical material and isolated the compound. DOT, HSAM and MHT were the coordinators and designed all the studies assays. RMX and MHT drafted the manuscript. All authors read and approved the final manuscript.

## Pre-publication history

The pre-publication history for this paper can be accessed here:

http://www.biomedcentral.com/1472-6882/12/139/prepub
